# Analysis of short-term clinical efficacy and immune function changes of advanced non-small cell lung cancer after radiotherapy or chemotherapy under CT-guided ^125^I seed implantation

**DOI:** 10.3389/fonc.2025.1667205

**Published:** 2025-11-06

**Authors:** Songbai Chen, Yunfeng Kou, Yuting Yuan, Guangsheng Zhao, Jun Zhou, Ruoyu Wang, Zhe Wang, Chuang Li

**Affiliations:** 1 Department of Radiology, Affiliated Zhongshan Hospital of Dalian University, Dalian, China; 2 Department of Intervention, Affiliated Zhongshan Hospital of Dalian University, Dalian, China; 3 Department of Medical Oncology, Affiliated Zhongshan Hospital of Dalian University, Dalian, China

**Keywords:** non-small cell lung cancer, ^125^I seed, clinical efficacy, peripheral T lymphocyte cells, cytokines

## Abstract

**Objective:**

To evaluate the clinical efficacy, safety, and changes in the immune status of advanced non-small cell lung cancer (NSCLC) patients with disease progression after chemoradiotherapy treated with CT-guided ^125^I radioactive seed implantation.

**Materials and methods:**

From January 2016 to June 2022, 34 NSCLC patients who progressed after radiotherapy and chemotherapy were studied retrospectively. There were 34 evaluable lesions, and ^125^I seeds were implanted into the lesions under CT guidance. The study’s endpoints were as follows: short-term clinical efficacy, quality of life score, and adverse reaction status assessment, with patients being collected for immune status assessment.

**Results:**

The average postoperative follow-up period was 16.58 ± 7.41 months. The 1-year postoperative survival rate was 76.47% (26/34), the 2-year postoperative survival rate was 58.82% (20/34), and the median overall survival was 16 (6–24) months (95% CI: 13.7–18.3). The 1-year progression-free survival (PFS) rate after the operation was 61.76% (21/34), the 2-year PFS rate was 41.18% (14/34), and the median PFS was 12.5 (1–24) months (95% CI: 10.8–16.2). Postoperative pneumothorax occurred in 11.76% of patients, minor bleeding in 5.88%, and pneumonia in 2.94%, all of which improved after symptomatic treatment. Compared with the preoperative results, the percentages of CD3^+^ and CD4^+^ T lymphocytes in the treatment group increased 1, 2, 3, and 6 months after surgery; the percentage of NK cells increased 3 and 6 months after surgery. The positive immune factor levels of IL-2 and TNF-α were increased at 2, 3, and 6 months after surgery; γ-IFN levels were increased at 1, 2, 3, and 6 months after surgery; IL-4 levels were decreased at 3 and 6 months after surgery; and IL-10 levels were decreased at 6 months after surgery. TH17 (IL-17) levels decreased at 1, 2, 3, and 6 months after surgery.

**Conclusion:**

CT-guided ^125^I particle therapy may be an effective treatment for NSCLC that has progressed following radiotherapy and chemotherapy. Local treatments improve patients’ quality of life and reduce tumor burden. CT-guided ^125^I radioactive seed implantation may improve the immune status of patients with recurrent or progressive NSCLC after radiotherapy and chemotherapy and may enhance the antitumor immune response.

## Introduction

Lung cancer is now the world’s second most common cancer (1), with non-small cell lung cancer accounting for 80%–85% of all cases (2). Some patients’ lesions are still progressing after chemoradiotherapy (3). Second-line chemotherapy has significant systemic toxicity and side effects that are often unbearable for patients (4). Because of the cumulative dose of the past, second radiotherapy is no longer appropriate for such patients (5), but CT-guided ^125^I radioactive seed implantation may be an appropriate treatment (6), which is currently being used to treat lung cancer (7). Unlike traditional radiotherapy, radioactive seed implantation can release γ-rays from the tumor tissue after seed implantation, causing DNA damage in tumor cells, leading to apoptosis; the range of ^125^I particles is 1.7 cm, and the dose distribution follows the inverse square law, increasing with decreasing distance and causing less damage to surrounding tissue (8, 9). It has been used to treat a variety of malignant tumors (10). It kills tumor cells by continuously emitting γ-rays, alters immune function, and regulates antitumor immunity (8, 9). Different treatments can cause changes in immune cells and factors. Chemotherapy generally impairs immune function. Traditional chemotherapy can inhibit tumor cell proliferation, but it also causes the depletion of the adaptive immune system and other defense mechanisms, such as lymphocyte depletion, bone marrow depletion, and the depletion of effector cells such as CD8^+^ and CD4^+^ (11). Although radiotherapy is considered to be the first-line treatment for advanced lung cancer, the immune response it causes is often characterized by a “double-edged sword”. Radiotherapy has been shown in studies to damage tumor cells and cause the immunogenic death of cancer cells. Dendritic cells (DCs) introduce these dead cancer cells to T cells in the draining lymph nodes. T cells play an important role in cellular immunity and induce antitumor immune responses (12). Furthermore, RT can suppress the immune environment, and the main drivers of immunosuppression are myeloid-derived suppressor cells (MDSCs), regulatory T cell (Tregs), and M2 macrophages, both of which can produce sperm, all of which can produce immunosuppressive factors, through the amidases, directly inhibiting antitumor CD4^+^ and CD8^+^ T cell activities. MDSCs inhibit T-cell function through the Jak/Stat pathway by increasing Reactive Oxygen Species (ROS) and Inducible Nitric Oxide Synthase (iNOS) levels, increasing T-cell apoptosis, and reducing MHC expression (13). This paper retrospectively studied the clinical efficacy and adverse reactions of 34 patients with advanced non-small cell lung cancer (NSCLC) who progressed after radiotherapy and chemotherapy using CT-guided ^125^I radioactive seed implantation at the Affiliated Zhongshan Hospital of Dalian University from January 2016 to June 2022. After treatment, we discovered that the immune status of patients after seeding had changed, so we collected data on the changes in the immune function of patients with advanced NSCLC who progressed after radiotherapy and chemotherapy from January 2020 to January 2022, before and after radioactive seed therapy. The synopsis is as follows.

## Materials and methods

### Research subjects

This study collected 44 patients with NSCLC who progressed after radiotherapy and chemotherapy and were admitted to our hospital’s Interventional Department from January 2016 to June 2022. This study was conducted in accordance with the Declaration of Helsinki and was approved by the Ethics Committee of the Affiliated Zhongshan Hospital of Dalian University (Approval Number: KY-2023-041-1). Applying the inclusion criteria, 34 cases met all the requirements, and pathology confirmed the initial diagnosis. There were 29 men and five women, aged 66.34 ± 10.72 years: 26 cases had TNM stage III, eight cases had IV stage, 18 (52.94%) cases had squamous cell carcinoma, and 16 cases had adenocarcinoma. Of the cases, 47.06% did not indicate radical surgery or had failed radiotherapy and chemotherapy before seed implantation surgery. Previously, only eight patients received radical external radiotherapy, 15 received only radical chemotherapy (the patients refused re-radiotherapy), and 11 received radical radiation therapy + chemotherapy. The local ethical agency reviewed and approved this study, and all patients provided signed informed consent before the operation (**Table 1**). Immunity was monitored in 28 of the 34 patients mentioned above, and the immune status of these patients was analyzed.

**Table 1:** General information of patients with non-small cell lung cancer.

Characteristic	Number of cases	Percentage
Gender		
Male	29	82.35
Female	5	17.65
Age (years)		
<60	12	35.29
>60	22	64.71
Pathological type		
Squamous cell carcinoma	18	52.94
Adenocarcinoma	16	47.06
Lesion location		
Surrounding type	12	35.29
Central type	22	64.71
TNM stage		
III	26	76.47
IV	8	23.53
Lesion diameter (cm)		
<3	6	17.65
3–5	17	50
>5	11	32.35
Previous treatment		
Radical radiotherapy	8	23.53
Radical chemotherapy	15	44.12
Radical (radiotherapy + chemotherapy)	11	32.35
Systemic treatment 6 months after operation		
Targeted therapy	10	29.41
Immunotherapy	6	17.65
Targeted therapy + immunotherapy	4	11.76
Other or none	14	41.18

The general situation of 34 patients with non-small cell lung cancer can be seen in terms of sex, age, pathological type, lesion location, stage, tumor diameter, previous treatment, and postoperative treatment. TNM, tumor, node, metastasis.

The inclusion criteria were as follows: 1) NSCLC patients who have progressed after first-line radiotherapy and chemotherapy and 2) have stage III or IV disease according to the Union for International Cancer Control (UICC) eighth edition TNM staging criteria and no indication of radical surgery. 3) The tumor was larger than 1.5 cm but smaller than 7.00 cm in diameter. 4) Karnofsky Performance Status (KPS) score was ≥ 70 points, with an expected survival time of more than 6 months. 5) The patients and their families agreed and provided signed informed consent. 6) Seed implantation therapy had no obvious contraindications. Exclusion criteria were as follows: 1) additional radiotherapy and chemotherapy following seed implantation; 2) poor overall health; 3) a serious organ disorder or failure in the heart, liver, kidneys, lungs, and other organs; and 4) those who did not have regular re-examinations 1, 2, 3, and 6 months after surgery.

### Instruments and equipment

Beijing Zhibo Hi-Tech Biotechnology Co., Ltd., provided radioactive ^125^I particles with radioactivity of 0.56–0.8 mCi, a diameter of 0.8 mm, a single particle length of 4.5 mm, a half-life of 59.6 days, a tissue penetration capacity of 1.7 cm, and a shell of nickel-titanium alloy cladding. Beijing Tianhang Kelin provided the particle implantation planning system, particle implantation positioning, and navigation system. The Mick particle implantation gun, Sinopharm Foreign Trade (Beijing) Co., Ltd., a CT analog positioning machine (Toshiba, Tokyo), and a flow detector (Becton Drive, Franklin Lakes, NJ, USA) were used.

## Method

### Preoperative preparation

Routine blood tests (coagulation function, liver and kidney function, and cardiopulmonary function) and other routine examinations were performed before the operation, as well as enhanced CT or MRI examinations to determine the location, extent, and surrounding structures of the tumor.

### Seed implantation strategy

The Treatment Planning System (TPS) planning system was used to calculate the tumor target dose, the number of implanted radioactive particles, and the placement site. The dosing algorithm was calculated according to the official report of the American Association of Physicists in Medicine (AAPM) (14–16). Imaging revealed that the tumor range was identified as the gross target volume (GTV), and the clinical target volume (CTV) was generated after 5 mm of external expansion. GTV and CTV dual-prescription doses were administered. The plan had to meet the requirements of the dual-prescription dose before it could be approved, and the organs at risk around the tumor had to be delineated. The planned preoperative D90GTV dose was 140 (110, 170) Gy, the CTV dose was 100 (70, 130) Gy, the median particle number was 35 (11, 132) particles, and the median dose activity was 0.6 (0.56, 0.8) mCi (**Table 2**).

**Table 2:** Seed implantation.

Seed implantation	Number
Number of ^125^I seeds	35 (11, 132)
Seed activity (mCI)	0.6 (0.56, 0.8)
D90 dose (Gy)	140 (110, 170)
6-month postoperative effect	
CR + PR	16 (47.05%)
SD + PD	18 (52.25%)
Survival rate	
1 year	26/34 (76.47%)
2 years	20/34 (58.85%)
Adverse reaction	
Grade I–II	10/34 (29.41%)
Grade III–IV	0

Particle implantation in 34 patients with non-small cell lung cancer and their recent efficacy.

CR, complete response; PR, partial response; SD, stable disease; PD, progressive disease.

### Intraoperative operations

Routine ECG monitoring was conducted during the operation. After a needle was placed under CT scan positioning, the ^125^I seeds were implanted according to the preoperative plan, ensuring no blood backflow, and the CT scan was reviewed to assess the operation quality. Supplemental seed implantation can be performed in the area to ensure the radiation dose requirement of the entire target area, observe for complications in the operation, and provide appropriate treatment.

### Postoperative treatment

Symptomatic treatment was routinely administered within 3 days of seed implantation, and CT was reviewed 3 days later to assess the condition of the operation area and the presence of complications. There was no statistically significant difference between D90 and V100 doses preoperatively and postoperatively. The postoperative GTV was 125 (95,155) Gy, and the postoperative CTV was 90 (60,120) Gy.

### Immune indicator detection

The percentages of CD3^+^ T cells, CD4^+^ T cells, CD8^+^ T cells, CD8^+^ T cells, NK cells, and cytokines TH1, TH2, and TH17 were measured from the peripheral blood of patients 3 days before and 3 days after surgery, and 1, 2, 3, and 6 months after surgery; the test data were analyzed using the FlowJo 7.6 software.

### Observation indicators

According to the Response Evaluation Criteria in Solid Tumors (RECIST) version 1.1, the changes in lesions before surgery and at 1, 3, and 6 months after surgery were evaluated to assess the local control effect (via CT or MRI scan). Specifically, the tumor response assessment was independently performed by two radiologists with more than 5 years of experience in thoracic oncology imaging, who were blinded to the patients’ clinical treatment information (including surgical details and postoperative adjuvant therapy) to avoid evaluation bias. In cases of inconsistent judgments between the two radiologists (e.g., discrepancies in defining partial response vs. stable disease), a third senior radiologist (with over 10 years of experience in oncology imaging) was invited to conduct a joint review of the images for arbitration, and the final consensus result was adopted as the evaluation outcome (17). The observation focus was the overall survival (OS) and disease-free stage [progression-free survival (PFS)], KPS score, and adverse reactions [acute radiation injury according to the Radiation Therapy Oncology Group/European Organization for Research and Treatment of Cancer (RTOG/EORTC) score as standard (18)]. The KPS score was used to assess the physical functional status of cancer patients (commonly applied in clinical practice and research to evaluate the functional capacity of patients with tumors).

### Statistical methods

The SPSS 23.0 statistical software was used for statistical analysis, and continuous variables that conformed to normal distribution were expressed as x ± s, while data that did not conform to normal distribution were expressed as M (min, max). General information was expressed as frequency (n) and percentage (%). Some graphs were created using GraphPad Prism 8.0.1.

## Result

### Clinical efficacy

Local lesions were monitored for 1, 3, and 6 months after the operation, with complete response (CR) in 1/34 at 1 month, partial response (PR) in 18/34, stable disease (SD) in 15/34, and progressive disease (PD) in 1/34. The local control rate (CR + PR + SD) was 97.05%, and the objective remission rate (CR + PR) was 55.88%; 3-month CR was achieved in 3/34 and PR in 13/34. The lesions were stable in 15/34 and progressed (PD) in 3/34, the local control rate (CR + PR + SD) was 91.17%, and the objective response rate (CR + PR) was 47.05% at 6 months. CR was achieved in 4/34, PR in 10/34, SD in 16/34, and PD in 4/34. The local control rate (CR + PR + SD) was 88.23%, and the objective response rate (CR + PR) was 41.17% (**Table 3**; **Supplementary Figure S1**).

**Table 3:** Clinical remission rate.

Months effect	1 month	3 months	6 months
CR	1	3	4
PR	18	13	10
SD	14	15	16
PD	1	3	4
DCR (%)	97.05	91.17	88.23
ORR (%)	55.88	47.05	41.17

Clinical efficacy, local control rate, and objective remission rate of 34 patients with non-small cell lung cancer 1, 3, and 6 months after operation. Objective Response Rate = (CR + PR + SD)/(CR + PR + SD + PD). Disease Control Rate = (CR + PR)/(CR + PR + SD + PD).

### Lesion diameter and KPS score

**Figure 1:**
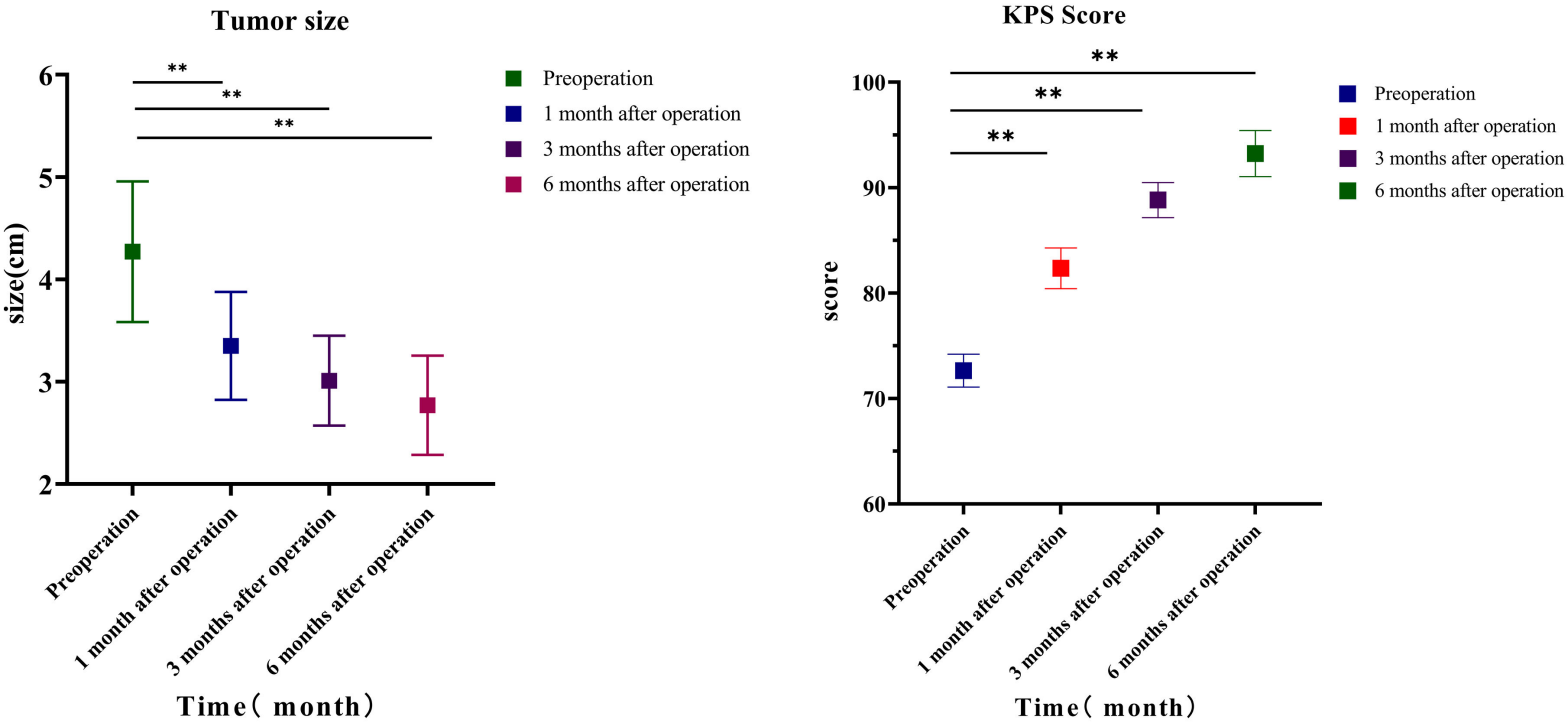
Lesion size and KPS. Comparison of tumor diameter of 34 patients with non-small cell lung cancer before operation and 1, 3, and 6 months after operation. KPS, Karnofsky Performance Status.

Comparing the changes in tumor size before the operation with those at 1, 3, and 6 months after the operation, it can be seen that the difference was statistically significant before surgery and 1 month after the operation (t = 5.502, *p* = 0.000004), 3 months after the operation (t = 5.45, *p* = 0.000005), 6 months after the operation (t = 5.474, *p* = 0.000005), 1 and 3 months after the operation (t = 2.257, *p* = 0.03), and 6 months after the operation (t = 2.4, *p* = 0.022); the change in tumor size between 3 and 6 months after the operation (t = 2.65, *p* = 0.097) had no statistical significance. The KPS score before the operation was 72.64 ± 4.47 points; KPS scores at 1, 3, and 6 months after the operation were 82.35 ± 5.53 points (t = 23.89, *p* < 0.001), 88.82 ± 4.77 points (t = 13.536, *p* < 0.001), and 93.23 ± 6.26 points (t = 6.84, *p* < 0.001), respectively. Comparing the changes in KPS before and 1, 3, and 6 months after the operation, it can be seen that before the operation and 1 month after the operation, t = −9.813, *p* < 0.0001; 3 months after the operation, t = 13.536, *p* < 0.001; and 6 months after the operation, t = 17.581, *p* < 0.001. The difference was statistically significant 1 and 3 months after the operation (t = 7.713, *p* < 0.0001), and 6 months after the operation, the difference was statistically significant (t = 10.423, *p* < 0.0001); the difference in KPS score between 3 and 6 months after the operation (t = 4.703, *p* < 0.0001) was statistically significant (**Table 4**; **Figure 1**).

**Table 4:** KPS score and lesion diameter.

Time	KPS score	Lesion diameter
Preoperative	72.6 ± 4.5	4.3 ± 2.0
One month after operation	82.4 ± 5.5**	3.4 ± 1.5**
Three months after operation	88.8 ± 4.8**	3.0 ± 1.3**
Six months after operation	93.2 ± 6.3**	2.8 ± 1.4**
F	148.977	13.367
*p*	<0.001	<0.001

Comparison of tumor diameter of 34 patients with non-small cell lung cancer before operation and 1, 3, and 6 months after operation. KPS, Karnofsky Performance Status. ** *p* < 0.001.

### Survival time

**Figure 2:**
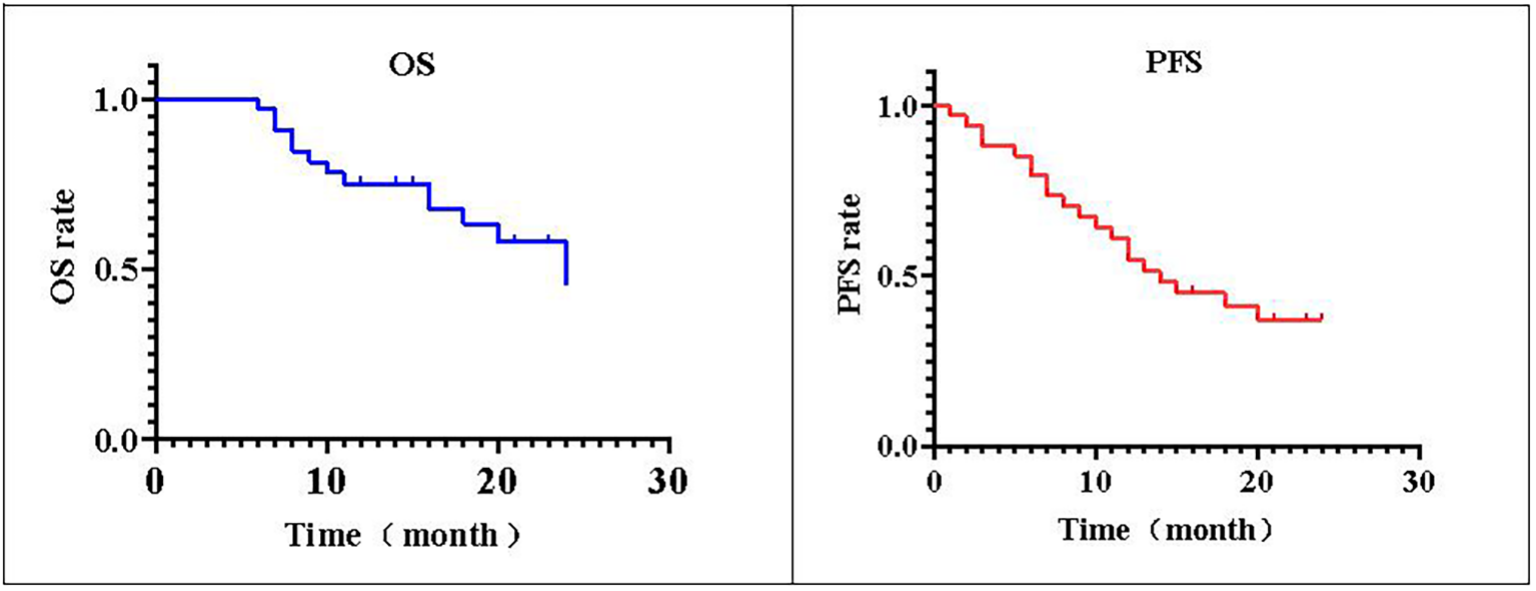
PFS and OS. The local progression-free survival (PFS) of 34 patients with non-small cell lung cancer is shown. The postoperative median progression-free survival time of patients was 76.47% (26/34). The survival rate 2 years after operation was 58.82% (20/34), and the median overall survival (OS) was 16 (6–24) months. The PFS rate was 61.76% (21/34) 1 year after operation and 41.18% (14/34) 2 years after operation.

The postoperative follow-up time was 16.58 ± 7.41 months. The 1-year postoperative survival rate was 76.47% (26/34), the 2-year postoperative survival rate was 58.82% (20/34), and the median OS was 16 (6–24) months (95% CI: 13.7–18.3). The 1-year PFS rate after the operation was 61.76% (21/34), the 2-year PFS rate was 41.18% (14/34), and the median PFS was 12.5 (1–24) months (95% CI: 10.8–16.2) (**Figure 2**).

### Side effects

#### Adverse reactions in the perioperative period

Postoperative pneumothorax in 10 cases, bleeding in two cases, and pneumonia in one case all improved after symptomatic treatment, and there were no adverse reactions such as massive bleeding, hemopneumothorax, or severe hemoptysis.

#### Particle-related adverse reactions

Perioperative adverse reactions included four cases of pneumothorax (grade 1), two cases of hemorrhage (grade 1), and one case of radiation-related acute pneumonia (grade 2). Particle-related adverse reactions included dyspnea in one case. Radiation-related esophageal reactions included dysphagia (grade 1) in two cases, leukopenia (grade 1) in three cases, and pneumonia (grade 1) in two cases, with no cardiotoxicity, central toxicity, mucosal hemorrhage, thrombocytopenia, etc. The radiation-related side effects are shown in **Table 5**.

**Table 5:** Information on complications.

Adverse reaction	Symptom	Case	Grade			Percentage (%)
			I	II	≥III	
Perioperative period	Pneumothorax	4	4	0	0	11.76
	Minimal hemorrhage	2	2	0	0	5.88
	Pneumonia	1	0	1	0	2.94
	Dyspnea	4	4	0	0	11.76
Treatment-associated reaction	Pneumonia	2	2	0	0	5.88
	Esophagus	2	2	0	0	5.88
	Skin	5	3	2	0	14.7
	Leukopenia	3	3	0	0	8.82

Particle postoperative adverse reactions in 34 patients with non-small cell lung cancer can be divided into perioperative adverse reactions and particle-related adverse reactions.

### Immune cells and factor changes

**Figure 3:**
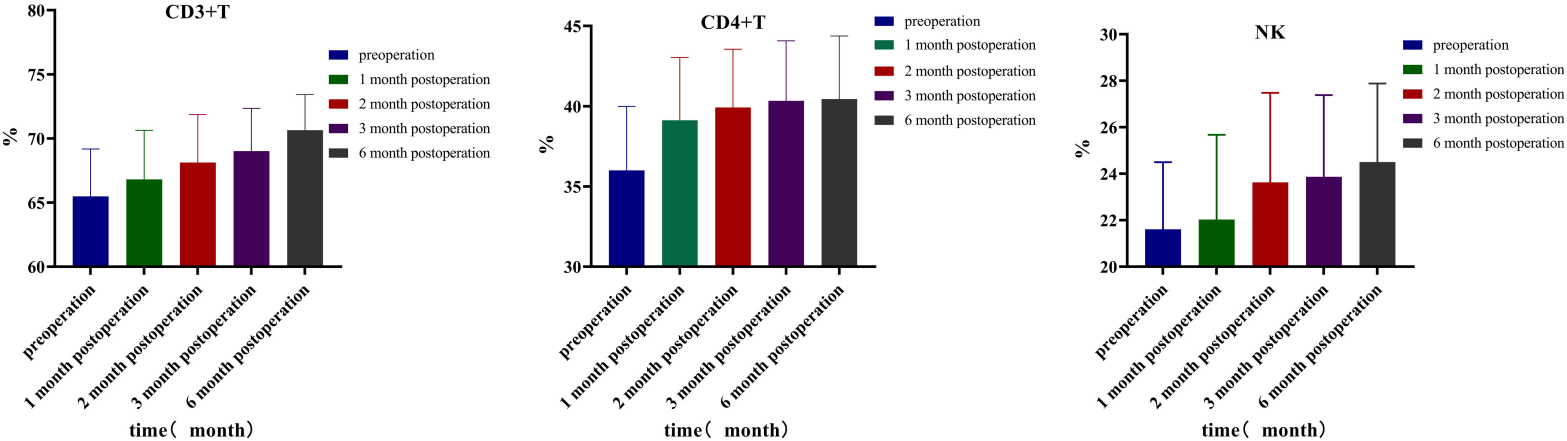
Lymphocyte subsets.

This study analyzed the changes in lymphocyte subsets 3 days before the operation and at 1, 2, 3, and 6 months after the operation. The results revealed that the percentage of CD3^+^ T cells before the operation was 65.48 ± 9.53. At 1, 2, 3, and 6 months after the operation, the percentages were 66.81 ± 9.86, 68.12 ± 9.67, 69.01 ± 8.59, and 70.65 ± 7.17, respectively. Compared to those before the operation, the percentages of CD3^+^ T cells gradually increased at 1 month (*p* = 0.009, 95% CI: −2.295, −0.368), 2 months (*p* < 0.001, 95% CI: −3.842, −1.445), 3 months (*p* < 0.001, 95% CI: −4.848, −2.214), and 6 months (*p* < 0.001, 95% CI: −6.747, −3.590) after the operation. The percentage of CD4^+^ T cells before the operation was 36.01 ± 10.27. At 1, 2, 3, and 6 months after the operation, the percentages increased to 39.13 ± 10.08 (*p* = 0.006, 95% CI: −5.256, −0.989), 39.92 ± 9.35 (*p* < 0.001, 95% CI: −5.846, −1.989), 40.34 ± 9.65 (*p* < 0.001, 95% CI: −6.077, −2.586), and 40.45 ± 10.12 (*p* < 0.001, 95% CI: −6.419, −2.460), respectively. The percentages of CD4^+^ T cells exhibited a gradual upward trend at 1, 2, 3, and 6 months after the operation. Three days before surgery, the NK cell percentage was 21.61 ± 7.45. At 1, 2, 3, and 6 months after the operation, the percentages increased to 22.03 ± 9.39 (*p* = 0.660, 95% CI: −2.380, 1.531), 23.63 ± 9.93 (*p* = 0.056, 95% CI: −4.092, 0.057), 23.87 ± 9.07 (*p* = 0.039, 95% CI: −4.399, −0.121), and 24.50 ± 8.71 (*p* = 0.014, 95% CI: −5.153, −0.642), respectively. Compared to pre-surgery levels, the NK cell percentages showed a gradual increase at 3 and 6 months after the operation. The percentage of CD8^+^ T lymphocytes on the first 3 days before surgery was 27.15 ± 10.72; at 1, 2, 3, and 6 months after surgery, they percentages were 26.53 ± 10.38 (*p* = 0.447, 95% CI: −1.034, 2.282), 26.67 ± 9.31 (*p* = 0.487, 95% CI: −0.911, 1.865), 26.58 ± 8.95 (*p* = 0.427, 95% CI: −0.875, 2.008), and 27.74 ± 10.45 (*p* = 0.479, 95% CI: −2.293, 1.105), respectively. CD8^+^ T lymphocytes exhibited a decreasing trend post-surgery and began to increase at 6 months post-surgery. Although the trend is noticeable, the difference is not statistically significant. The CD4^+^/CD8^+^ ratio was 1.66 ± 1.11 three days before surgery; it was 1.81 ± 1.40 (*p* = 0.304, 95% CI: −0.448, 0.145), 1.77 ± 1.20 (*p* = 0.294, 95% CI: −0.343, 0.108), 1.75 ± 0.97 (*p* = 0.231, 95% CI: −0.251, 0.063), and 1.75 ± 1.13 (*p* = 0.314, 95% CI: −0.288, 0.096) at 1, 2, 3, and 6 months after surgery, respectively. Although the CD4^+^/CD8^+^ ratio exhibited an increasing trend post-surgery, the difference was not statistically significant (**Table 6**, **Figure 3**).

**Table 6:** Detection value of lymphocyte subsets in different time periods.

Time	CD3^+^ cell percentage (%)	CD4^+^ cell percentage (%)	CD8^+^ cell percentage (%)	CD4^+^/CD8^+^	NK cell percentage (%)
Preoperative	65.48 ± 9.53	36.01 ± 10.27	27.15 ± 10.72	1.66 ± 1.11	21.61 ± 7.45
One month after operation	66.81 ± 9.86**	39.13 ± 10.08**	26.53 ± 10.38	1.81 ± 1.40	22.03 ± 9.39
Two months after operation	68.12 ± 9.67***	39.92 ± 9.35***	26.67 ± 9.31	1.77 ± 1.20	23.63 ± 9.93
Three months after operation	69.01 ± 8.59***	40.34 ± 9.65***	26.58 ± 8.95	1.75 ± 0.97	23.87 ± 9.07*
Six months after operation	70.65 ± 7.17***	40.45 ± 10.12***	27.74 ± 10.45	1.75 ± 1.13	24.50 ± 8.71*
F	14.1719	6.2478	0.5149	0.3671	3.5234
*p*	<0.001	0.001	0.725	0.8296	0.021

* *p* < 0.05; ** *p* < 0.01; *** *p* < 0.001.

**Figure 4:**
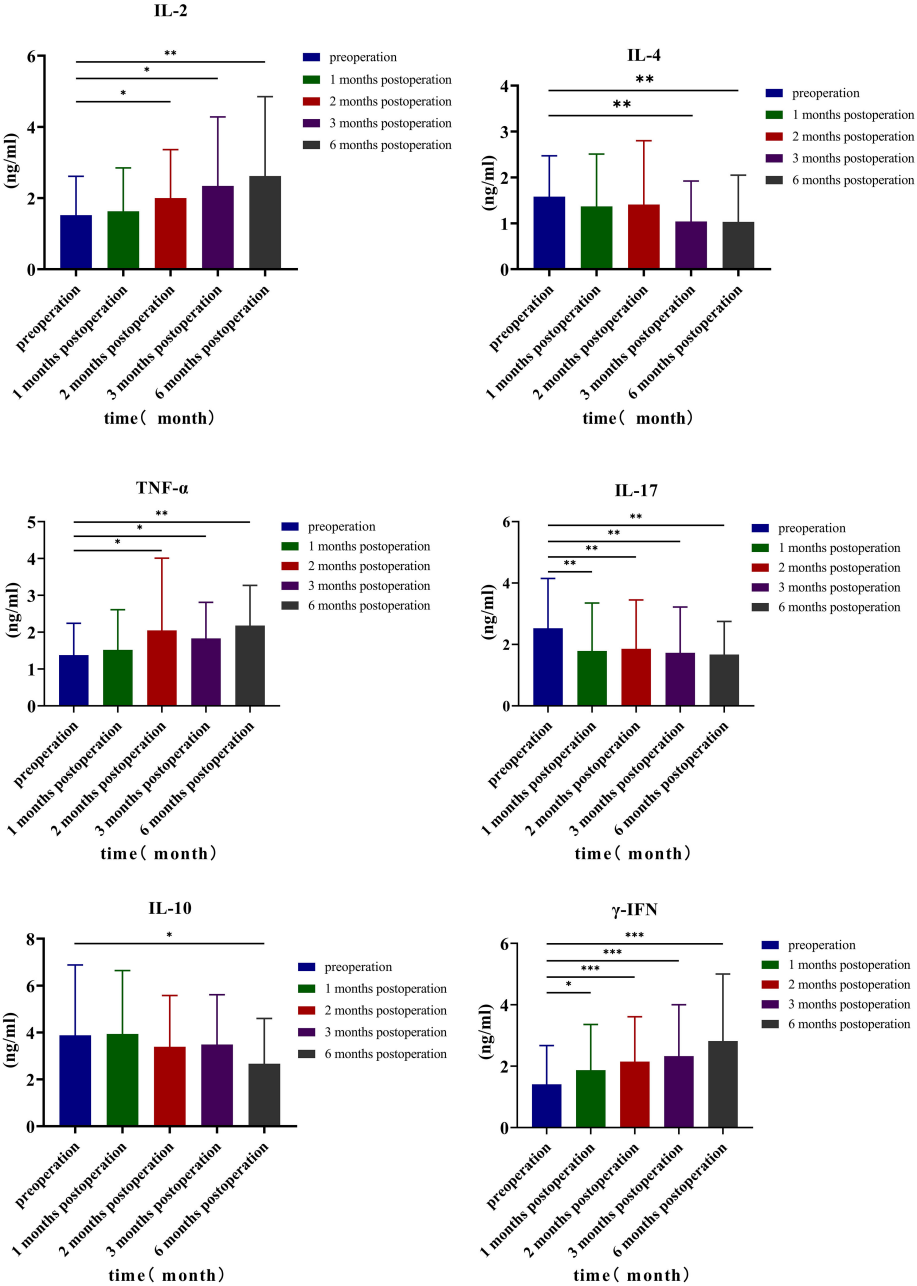
TH1, TH2, and TH17 cytokines.

Analyzing the changes in TH1, TH2, and TH17 levels 3 days before surgery and at 1, 2, 3, and 6 months after surgery, the results indicated that the preoperative IL-2 level was 1.52 ± 1.09 ng/mL; at 1, 2, 3, and 6 months after surgery, the levels were 1.63 ± 1.22, 2.00 ± 1.36, 2.34 ± 1.94, and 2.62 ± 2.23 ng/mL, respectively. Comparing the preoperative levels with those at 2 months (*p* = 0.049, 95% CI: −0.964, −0.001), 3 months (*p* = 0.025, 95% CI: −1.531, −0.111), and 6 months after surgery (*p* = 0.009, 95% CI: −1.896, −0.292), it was observed that the level of IL-2 increased, and the differences were statistically significant. The preoperative TNF-α level was 1.38 ± 0.86 ng/mL; the levels increased to 1.52 ± 1.09, 2.05 ± 1.96, 1.83 ± 0.98, and 2.18 ± 1.09 ng/mL at 1, 2, 3, and 6 months after surgery, respectively. Compared with the preoperative level, the TNF-α levels increased significantly at 2 months (*p* = 0.038, 95% CI: −1.298, −0.041), 3 months (*p* = 0.033, 95% CI: −0.858, −0.040), and 6 months after surgery (*p* < 0.001, 95% CI: −1.201, −0.3990), with statistically significant differences. Similarly, the preoperative γ-IFN level was 1.41 ± 1.26 ng/mL; the levels increased to 1.87 ± 1.49, 2.15 ± 1.46, 2.33 ± 1.67, and 2.82 ± 2.18 (ng/mL) at 1, 2, 3, and 6 months after the operation, respectively. Compared with the preoperative level, the γ-IFN levels increased significantly at 1 month (*p* = 0.023, 95% CI: −0.840, −0.068), 2 months (*p* < 0.001, 95% CI: −1.092, −0.376), 3 months (*p* < 0.001, 95% CI: −1.349, −0.477), and 6 months after surgery (*p* < 0.001, 95% CI: −2.064, −0.751), with statistically significant differences. Compared with preoperative levels, IL-4 levels decreased at 3 months (*p* = 0.004, 95% CI: 0.187, 0.905) and 6 months (*p* = 0.008, 95% CI: 0.154, 0.948) after the operation, while IL-10 levels decreased at 6 months after the operation (*p* = 0.026, 95% CI: 0.156, 2.276), with statistical significance. The preoperative level of TH17 (IL-17) was 2.53 ± 1.62 ng/mL, and at 1, 2, 3, and 6 months after the operation, the levels were 1.79 ± 1.56, 1.86 ± 1.59, 1.73 ± 1.49, and 1.67 ± 1.08 ng/mL, respectively. Compared with preoperative and postoperative levels at 1 month (*p* = 0.003, 95% CI: 0.268, 1.208), 2 months (*p* = 0.008, 95% CI: 0.186, 1.150), 3 months (*p* = 0.003, 95% CI: 0.292, 1.299), and 6 months (*p* = 0.003, 95% CI: 0.292, 1.299), IL-17 values decreased, and the difference was statistically significant (**Table 7**, **Figure 4**).

**Table 7:** Detection value of TH1, TH2, and TH17 cytokines in different time periods.

	IL-2	TNF	IFN-γ	IL-4	IL-6	IL-10	IL-17
Preoperative	1.52 ± 1.09	1.58 ± 0.89	19.42 ± 15.65	3.88 ± 3.00	1.38 ± 0.86	1.41 ± 1.26	2.53 ± 1.62
One month after operation	1.63 ± 1.22	1.37 ± 1.14	21.59 ± 17.15	3.94 ± 2.70	1.52 ± 1.09	1.87 ± 1.49*	1.79 ± 1.56**
Two months after operation	2.00 ± 1.36*	1.41 ± 1.39	21.06 ± 14.75	3.39 ± 2.19	2.05 ± 1.96*	2.15 ± 1.46***	1.86 ± 1.59**
Three months after operation	2.34 ± 1.94*	1.04 ± 0.88**	19.02 ± 13.83	3.49 ± 2.12	1.83 ± 0.98*	2.33 ± 1.67***	1.73 ± 1.49**
Six months after operation	2.62 ± 2.23**	1.03 ± 1.02**	17.57 ± 13.33	2.67 ± 1.93*	2.18 ± 1.09**	2.82 ± 2.18***	1.67 ± 1.08**
F	2.86	2.93	1.79	4.08	7.06	7.12	3.08
*p*	0.045	0.042	0.163	0.012	0.001	0.001	0.035

* *p* < 0.05; ** *p* < 0.01; *** *p* < 0.001.

## Discussion

Disease progression in patients with NSCLC following chemoradiotherapy remains a major clinical challenge at present. According to some studies, approximately 40% of patients experience local progression or recurrence after concurrent chemoradiotherapy (19). Although immunotherapy has emerged as a new treatment method for NSCLC in recent years, drug resistance has also developed (20). Local progression remains an important clinical problem. Studies have shown that the time to local control and OS in patients with stage III NSCLC who relapsed after chemoradiotherapy and salvage surgery was 10–22 months and 13–76 months, respectively (21). The 5-year overall survival rate was 44.7% (22). Some studies have revealed that after re-radiation with intensity-modulated radiation therapy and proton beam therapy (PBT), the 2-year OS was 30%–40%, and the rate of ≥grade 3 pulmonary toxicity was 0%–20% (23). According to another study, the rate of ≥grade 3 toxicity after re-radiotherapy (PBT) can reach 42% (24). Whether the second-line chemotherapy is single-agent or combined chemotherapy, the median local control rate was only 2.7–4.5 months, and the 1-year average survival rate ranged from 24.8% and 52.7% (25). Therefore, in patients with advanced NSCLC who continue to progress or relapse after radiotherapy and chemotherapy, there is an urgent need for a treatment method with fewer adverse effects to effectively control local lesions.

In recent years, the concept of precision medicine has gradually entered clinicians’ minds, and radioactive particle brachytherapy has been used in the treatment of various solid tumors due to its unique advantages. There are significant differences in the immune effects between brachytherapy and external beam radiotherapy (EBRT). Brachytherapy, via localized high-dose irradiation, is more likely to induce tumor cell apoptosis and release a large number of tumor-associated antigens (TAAs), activating specific antitumor immunity. Additionally, its minimal damage to normal tissues reduces inflammation-mediated immune suppression. In contrast, EBRT has a broad dose distribution; although it can trigger a systemic immune response, extensive irradiation of normal tissues tends to exacerbate immune suppression, weakening the antitumor immune effect. Given the continuous emission of low-dose gamma rays and continuous damage to tumor cells, the scope of action is only 1.7 cm, so in theory, as long as the target area is planned before surgery, the damage to surrounding organs is minimal (26). It is currently being used to treat lung cancer. Wang reported that the median survival time after ^125^I treatment for NSCLC patients was 395 days. The 1-year survival rate was approximately 78.1%, the 2-year survival rate was approximately 56.1%, and the complication rate was approximately 14.1% (27). Huo studied 38 cases of locally recurrent NSCLC after seed implantation and discovered that the 2-month local control rate was 92%, and the 2-year PFS%, Local Control Rate (LCR), and OS% were 39.5%, 83.5%, and 83.5%, respectively. Of the cases, 47.4% had only minor complications that were relieved after symptomatic treatment (28). The studies mentioned above have confirmed that radioactive ^125^I seed implantation as a local treatment method has a particular clinical effect on recurrent or progressive NSCLC. In this study, at 1, 3, and 6 months after surgery, the local control rates were 97.05%, 91.17%, and 88.23%, respectively, and the size of the lesions was 3.35 ± 1.51, 3.01 ± 1.26, and 2.77 ± 1.5 cm, respectively. A 1.39-cm lesion gradually shrank after surgery, and the 1-year and 2-year survival rates were 76.47% and 58.82%, respectively, which were consistent with the previous research findings and indicated that radioactive particles had a positive effect on local tumor control. It is worth noting that most patients in this study received adjuvant systemic therapies (such as immune checkpoint inhibitors, targeted therapy, or salvage chemotherapy) before or after seed implantation; these systemic treatments may have synergistically contributed to the favorable survival outcomes by controlling potential distant micro-metastases and enhancing the antitumor immune response, thereby complementing the local control effect of radioactive seeds. Toxicity and side effects have always been barriers to treating recurrent NSCLC. Wang et al. reported that ^125^I radioactive particles were used to treat NSCLC after first-line chemotherapy failed. When compared to second-line chemotherapy, ^125^I brachytherapy improved long-term quality of life and had fewer adverse reactions (6). In this study, perioperative adverse reactions were predominantly postoperative pneumothorax in four cases, followed by hemorrhage and acute pneumonia; particle-related adverse reactions included dyspnea, radiation-related esophageal reactions, leukopenia, and pneumonia; all were grade 1–2 adverse reactions that improved after symptomatic treatment. It is clear that the safety of ^125^I seed implantation is high, with a low incidence of postoperative adverse reactions.

Changes in lymphocyte subsets are important in antitumor responses: lymphocytes are thought to be the primary effector cells in tumor immunity, participating in tumor microenvironment formation and regulating local tumor immunity. It mainly includes T cells, B cells, and NK cells. T cells are classified as CD3^+^, CD4^+^, or CD8^+^. Total T lymphocytes (CD3) are immune functional cells that directly reflect the activity and number of immune functional cells involved in the immune response. CD4^+^ helper T lymphocytes (Th) are essential components of effector T cells (29). CD4^+^ T cells are divided into four subsets: helper T cells type 1 (TH1) and type 2 (TH2) are the two main classes of CD4^+^ T helper cells, each with distinct subsets (30). Each subset can secrete different cytokines; Th1 cells primarily secrete interferon-γ (IFN-γ), IL-2, and tumor necrosis factor-alpha (TNF-α); activated Th1 cells and the cytokines that they secrete have a strong antitumor activity and immunomodulatory effect and can play a positive regulatory role in the tumor immune microenvironment. IL-2 can stimulate the antitumor activity of NK^+^ cells and promote macrophage M1 polarization through the Jak3–Stat pathway (31); TNF-α can directly cause tumor cell apoptosis, break tumor cell DNA, cause cell shrinkage and death, and activate antitumor immunity by affecting natural killer and CD8^+^ T cells (30, 32). Th2 cells primarily secrete IL-4, IL-6, and IL-10; IL-4 regulates MDSCs by synthesizing arginase 1 (Arg1) and decomposing l-arginine to induce T-cell apoptosis and tumor immunosuppressive effects (33). Li et al. reported that lymphopenia, CD3^+^, CD3^+^CD4^+^, CD3^+^CD8^+^ T lymphocytes, B lymphocytes, NK cells, and CD4^+^/CD8^+^ ratios were lower in patients with NSCLC who received radiotherapy (34). Chen et al. reported that when compared to baseline, after chemoradiotherapy, the proportions of CD3^+^ T cells (*p* < 0.001), CD8^+^ T cells (*p* < 0.001), and CD3^+^CD56^+^ NKT cells (*p* = 0.025) increased, while the percentages of CD4^+^ T cells (*p* < 0.001), CD4/CD8^+^ ratio (*p* < 0.001), CD19^+^ B cells (*p* < 0.001), and CD3^−^CD56^+^ NK cells (*p* < 0.001) decreased (35). There is currently no research on the changes in immune status following particle surgery. Compared with the preoperative results, the percentages of CD3^+^ and CD4^+^ T lymphocytes in the treatment group increased 1, 2, 3, and 6 months after surgery, and the percentages of NK cells increased 3 and 6 months after surgery. The positive immune factor levels of IL-2 and TNF-α were increased at 2, 3, and 6 months after surgery; γ-IFN levels were increased at 1, 2, 3, and 6 months after surgery; IL-4 levels were decreased at 3 and 6 months after surgery; and IL-10 levels were decreased at 6 months after surgery. TH17 (IL-17) levels decreased at 1, 2, 3, and 6 months after surgery. Changes in the body’s immune status can regulate the immune response. The above findings show that radioactive particles may alter the immune function of the body at various times after surgery and boost patients’ antitumor immunity in the short term.

## Conclusion

We believe that after radiotherapy and chemotherapy, CT-guided ^125^I seed implantation may be an effective treatment for recurrent or progressive NSCLC and that it can be used as one of the local treatments for reducing local tumor burden and improving patients’ quality of life. In addition, CT-guided ^125^I seed implantation may improve the immune status of NSCLC patients and regulate the body’s immune function. Furthermore, this study has some limitations, such as a small number of cases, a short follow-up period, and a retrospective single-arm study. Therefore, further larger-sample prospective, multi-center trials are required to verify the efficacy and immune function changes of CT-guided ^125^I seed implantation in the treatment of recurrent or advanced NSCLC after radiotherapy and chemotherapy.

## Data Availability

The raw data supporting the conclusions of this article will be made available by the authors, without undue reservation.

## Supplementary material

Supplementary Figure 1 Representative Images of CT-Guided ¹²^5^I Seed Implantation and Lesion Changes Before and After Treatment. **Supplementary Figure S1** a typical case. **(A)** Preoperative Enhanced Computed Tomography (CT) **(B)** Simulation plan before 125 I brachytherapy **(C)** Intraoperative Findings of CT-Guided 125 I brachytherapy **(D)** Non-Contrast Lung CT focusing on NSCLC 3 days after 125 I brachytherapy **(E)** Non-Contrast Lung CT focusing on NSCLC 60 days after 125I brachytherapy **(F)** Non-Contrast Lung CT focusing on NSCLC 180 days after 125 I brachytherapy.

## References

[1] Sung H Ferlay J Siegel RL Laversanne M Soerjomataram I Jemal A . Global cancer statistics 2020: GLOBOCAN estimates of incidence and mortality worldwide for 36 cancers in 185 countries. CA: Cancer J Clin. (2021) 71:209–49. doi: 10.3322/caac.21660, PMID: 33538338

[2] Jia Z . Interpretation of updated points of 2021 CSCO guideline for non-small-cell lung cancer. J J Pract Oncol. (2022) 37:8–15. doi: 10.1080/10463356.2022.2030612

[3] Wu C Li B Sun G Peng C Xiang D . Efficacy and safety of iodine-125 brachytherapy combined with chemotherapy in the treatment of advanced NSCLC in the elderly. OncoTargets Ther. (2018) 11:6617–24. doi: 10.2147/OTT.S174457, PMID: 30349295 PMC6188210

[4] Alexander M Kim SY Cheng H . Update 2020: management of non-small cell lung cancer. Lung. (2020) 198:897–907. doi: 10.1007/s00408-020-00407-5, PMID: 33175991 PMC7656891

[5] Bolke E Matuschek C . Images in clinical medicine. Radiation pneumonitis after radiotherapy for breast cancer. New Engl J Med. (2009) 361:e65. doi: 10.1056/NEJMicm0810650, PMID: 20042752

[6] Wang H Lu J Zheng XT Zha JH Jing WD Wang Y . Oligorecurrence non-small cell lung cancer after failure of first-line chemotherapy: computed tomography-guided (125)I seed implantation vs. second-line chemotherapy. Front Oncol. (2020) 10:470. doi: 10.3389/fonc.2020.00470, PMID: 32373512 PMC7179670

[7] Dai F Wang J An H Lei T Tang K Ma X . Therapy of (125)I particles implantation inhibited the local growth of advanced non-small cell lung cancer: a retrospective clinical study. Am J Transl Res. (2019) 11:3737–49. doi: 10.11655/ajtr.2019.11.3737, PMID: 31312384 PMC6614640

[8] Itami J . Modern development of high-dose-rate brachytherapy. Japanese J Clin Oncol. (2020) 50:490–501. doi: 10.1093/jjco/hyaa029, PMID: 32134450

[9] He P Guan S Ren E Chen H Chen H Peng Y . Precision interventional brachytherapy: A promising strategy toward treatment of Malignant tumors. Front Oncol. (2021) 11:753286. doi: 10.3389/fonc.2021.753286, PMID: 34692537 PMC8531520

[10] Xue H Qiu B Wang H Jiang P Sukocheva O Fan R . Stereotactic ablative brachytherapy: recent advances in optimization of radiobiological cancer therapy. Cancers. (2021) 13:3493. doi: 10.3390/cancers13143493, PMID: 34298703 PMC8304109

[11] Pusuluri A Wu D Mitragotri S . Immunological consequences of chemotherapy: Single drugs, combination therapies and nanoparticle-based treatments. J Controlled Release: Off J Controlled Release Soc. (2019) 305:130–54. doi: 10.1016/j.jconrel.2019.04.020, PMID: 31004668

[12] Demaria S Golden EB Formenti SC . Role of local radiation therapy in cancer immunotherapy. JAMA Oncol. (2015) 1:1325–32. doi: 10.1001/jamaoncol.2015.2756, PMID: 26270858

[13] Oweida A Paquette B . Reconciling two opposing effects of radiation therapy: stimulation of cancer cell invasion and activation of anti-cancer immunity. Int J Radiat Biol. (2021) 97:1–13. doi: 10.1080/09553002.2021.1910377, PMID: 34264178

[14] Nath R Anderson LL Luxton G Weaver KA Williamson JF Meigooni AS . Dosimetry of interstitial brachytherapy sources: recommendations of the AAPM Radiation Therapy Committee Task Group No. 43. American Association of Physicists in Medicine. Med Phys. (1995) 22:209–34. doi: 10.1118/1.597458, PMID: 7565352

[15] Yu Y Anderson LL Li Z Mellenberg DE Nath R Schell MC . Permanent prostate seed implant brachytherapy: report of the American Association of Physicists in Medicine Task Group No. 64. Med Phys. (1999) 26:2054–76. doi: 10.1118/1.598721, PMID: 10535622

[16] Rivard MJ Coursey BM DeWerd LA Hanson WF Huq MS Ibbott GS . Update of AAPM Task Group No. 43 Report: A revised AAPM protocol for brachytherapy dose calculations. Med Phys. (2004) 31:633–74. doi: 10.1118/1.1646040, PMID: 15070264

[17] Nishino M . Tumor response assessment for precision cancer therapy: response evaluation criteria in solid tumors and beyond. Am Soc Clin Oncol Educ Book Am Soc Clin Oncol Annu Meeting. (2018) 38:1019–29. doi: 10.1200/EDBK_201441, PMID: 30231378

[18] Mao MH Zhang J Zhang JG . Comparing the RTOG/EORTC and LENT-SOMA scoring systems for the evaluation of late skin toxicity after (125)I seed brachytherapy for parotid gland cancer. Brachytherapy. (2018) 17:250. doi: 10.1016/j.brachy.2017.11.017, PMID: 29406124

[19] Agbarya A Shalata W Addeo A Abu-Hashim H Khoury T El-Hajj M . Real-world journey of unresectable stage III NSCLC patients: current dilemmas for disease staging and treatment. J Clin Med. (2022) 11:1738. doi: 10.3390/jcm11061738, PMID: 35330063 PMC8949111

[20] Prasad RN Williams TM . A narrative review of toxicity of chemoradiation and immunotherapy for unresectable, locally advanced non-small cell lung cancer. Transl Lung Cancer Res. (2020) 9:2040–50. doi: 10.21037/tlcr-20-638, PMID: 33209624 PMC7653152

[21] Hamada A Soh J Mitsudomi T . Salvage surgery after definitive chemoradiotherapy for patients with non-small cell lung cancer. Transl Lung Cancer Res. (2021) 10:555–62. doi: 10.21037/tlcr-20-453, PMID: 33569336 PMC7867739

[22] Kobayashi AK Nakagawa K Nakayama Y Ohe Y Yotsukura M Uchida S . Salvage surgery compared to surgery after induction chemoradiation therapy for advanced lung cancer. Ann Thorac Surg. (2021) 112:1996–2003. doi: 10.1016/j.athoracsur.2021.10.036, PMID: 34843695

[23] Yang K Suh YG Shin H Pyo H Moon SH Ahn YC . Toxicity of proton therapy versus photon therapy on salvage re-irradiation for non-small cell lung cancer. Life (Basel Switzerland). (2022) 12:292. doi: 10.3390/life12020292, PMID: 35207579 PMC8876714

[24] Vyfhuis MAL Rice S Remick J Mossahebi S Badiyan S Mohindra P . Reirradiation for locoregionally recurrent non-small cell lung cancer. J Thorac Dis. (2018) 10:S2522–s2536. doi: 10.21037/jtd.2017.12.50, PMID: 30206496 PMC6123190

[25] Gridelli C Baas P Barlesi F Ciardiello F Crinò L Felip E . Second-line treatment options in non-small-cell lung cancer: report from an international experts panel meeting of the Italian association of thoracic oncology. Clin Lung Cancer. (2018) 19:301–14. doi: 10.1016/j.cllc.2017.12.010, PMID: 29396237

[26] Wang J Chai S Wang R Zheng G Zhang K Huo B . Expert consensus on computed tomography-assisted three-dimensional-printed coplanar template guidance for interstitial permanent radioactive (125)I seed implantation therapy. J Cancer Res Ther. (2019) 15:1430–4. doi: 10.4103/jcrt.JCRT_434_19, PMID: 31939420

[27] Wang Y Zhu L Lin X He C An Z Tang J . Therapeutic effect of CT-guided ¹²^5^I seed implantation on advanced lung cancer and pulmonary metastatic carcinoma. Zhongguo fei ai za zhi = Chin J Lung Cancer. (2020) 23:424–8. doi: 10.3779/j.issn.1009-3419.2020.103.04, PMID: 32517444 PMC7309549

[28] Huo X Wang H Yang J Li X Yan W Huo B . Effectiveness and safety of CT-guided (125)I seed brachytherapy for postoperative locoregional recurrence in patients with non-small cell lung cancer. Brachytherapy. (2016) 15:370–80. doi: 10.1016/j.brachy.2016.02.001, PMID: 26944267

[29] Chraa D Naim A Olive D Badou A . T lymphocyte subsets in cancer immunity: Friends or foes. J Leukocyte Biol. (2019) 105:243–55. doi: 10.1002/JLB.MR0318-097R, PMID: 30387907

[30] Basu A Ramamoorthi G Albert G Gallen C Beyer A Snyder C . Differentiation and regulation of T(H) cells: A balancing act for cancer immunotherapy. Front Immunol. (2021) 12:669474. doi: 10.3389/fimmu.2021.669474, PMID: 34012451 PMC8126720

[31] Wenjing Q Yan L Yu W ailian Y . IL-2 promotes macrophage M1 polarization *via* the Jak3-Stat5 signal pathway. Basic Clin Med. (2015) 35:1055–60.

[32] Sheng Y Li F Qin Z . TNF receptor 2 makes tumor necrosis factor a friend of tumors. Front Immunol. (2018) 9:1170. doi: 10.3389/fimmu.2018.01170, PMID: 29892300 PMC5985372

[33] Ito SE Shirota H Kasahara Y Saijo K Ishioka C . IL-4 blockade alters the tumor microenvironment and augments the response to cancer immunotherapy in a mouse model. Cancer Immunol Immunother: CII. (2017) 66:1485–96. doi: 10.1007/s00262-017-2043-6, PMID: 28733709 PMC11029029

[34] Li H Yu H Lan S Zhao D Liu Y Cheng Y . Aberrant alteration of circulating lymphocyte subsets in small cell lung cancer patients treated with radiotherapy. Technol Cancer Res Treat. (2021) 20:15330338211039948. doi: 10.1177/15330338211039948, PMID: 34851203 PMC8649432

[35] Chen Y Jin Y Hu X Chen M . Effect of chemoradiotherapy on the proportion of circulating lymphocyte subsets in patients with limited-stage small cell lung cancer. Cancer Immunol Immunother. (2021) 70:2867–76. doi: 10.1007/s00262-021-02902-x, PMID: 33674986 PMC10991102

