# Supplementary figures and images for: Analysis of short-term clinical efficacy and immune function changes of advanced non-small cell lung cancer after radiotherapy or chemotherapy under CT-guided 125I seed implantation
Source: Front Oncol. 2025 Nov 6;15:1667205. doi: 10.3389/fonc.2025.1667205 (PMC12631448; doi:10.3389/fonc.2025.1667205)

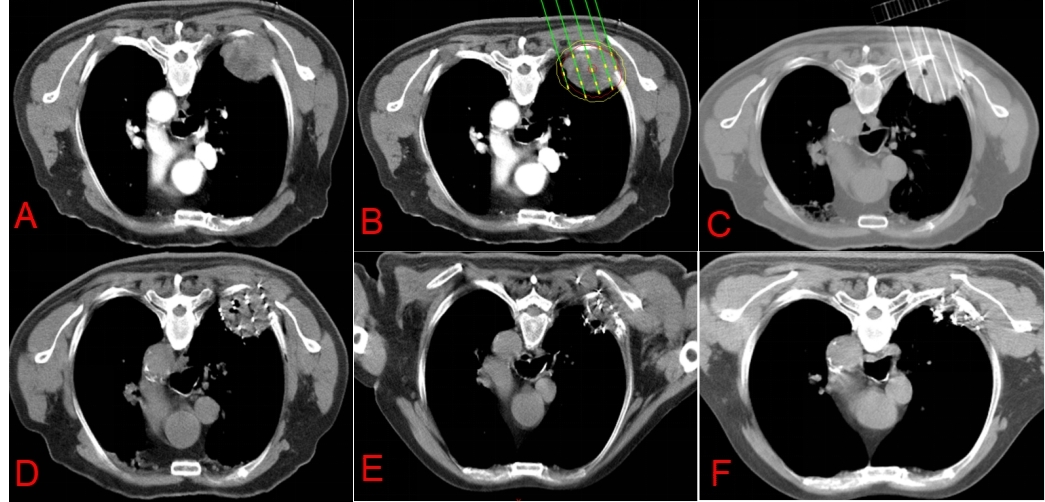

Supplement: Supplementary Figure 1 — Representative Images of CT-Guided ¹²5I Seed Implantation and Lesion Changes Before and After Treatment. Supplementary Figure S1 a typical case. (A) Preoperative Enhanced Computed Tomography (CT) (B) Simulation plan before 125 I brachytherapy (C) Intraoperative Findings of CT-Guided 125 I brachytherapy (D) Non-Contrast Lung CT focusing on NSCLC 3 days after 125 I brachytherapy (E) Non-Contrast Lung CT focusing on NSCLC 60 days after 125I brachytherapy (F) Non-Contrast Lung CT focusing on NSCLC 180 days after 125 I brachytherapy. [file Image1.jpg]
